# The biophysics of water in cell biology: perspectives on a keystone for both marine sciences and cancer research

**DOI:** 10.3389/fcell.2024.1403037

**Published:** 2024-05-13

**Authors:** Daniel L. Pouliquen

**Affiliations:** Inserm, CNRS, CRCI^2^NA, Nantes Université, University of Angers, Angers, France

**Keywords:** water, biophysics, water-protein interactions, water channels, proton transport, membranes, marine biology, deep-sea biology

## Abstract

The biophysics of water, has been debated over more than a century. Although its importance is still underestimated, significant breakthroughs occurred in recent years. The influence of protein condensation on water availability control was documented, new findings on water-transport proteins emerged, and the way water molecules rearrange to minimize free energy at interfaces was deciphered, influencing membrane thermodynamics. The state of knowledge continued to progress in the field of deep-sea marine biology, highlighting unknown effects of high hydrostatic pressure and/or temperature on interactions between proteins and ligands in extreme environments, and membrane structure adaptations. The role of osmolytes in protein stability control under stress is also discussed here in relation to fish egg hydration/buoyancy. The complexity of water movements within the cell is updated, all these findings leading to a better view of their impact on many cellular processes. The way water flow and osmotic gradients generated by ion transport work together to produce the driving force behind cell migration is also relevant to both marine biology and cancer research. Additional common points concern water dynamic changes during the neoplastic transformation of cells and tissues, or embryo development. This could improve imaging techniques, early cancer diagnosis, and understanding of the molecular and physiological basis of buoyancy for many marine species.

## 1 Introduction

Water is essential to the biosphere, shaping the earth and making life possible, where alteration of oceanic crust and serpentinization process play fundamental roles (Brovarone et al., 2020; Andreani et al., 2023; Schwander et al., 2023). In addition to being the medium of biology, water also represents the main actor in metabolism (Frenkel-Pinter et al., 2021). The biophysics of water, a central question for both marine biology and biomedical sciences, is a potential transdisciplinarybridge, and after more than a century of investigations, it is still receiving increasing attention, raising questions and perspectives. The anomalous properties of liquid water, continuously debated with, are still challenged (Urbic and Dill, 2018). In cell biology, the importance of water has always been underestimated, however its role in protein folding and structure, proton and electron transfer, nucleic structure, and communication at a distance were emphasized (Chaplin, 2006). Given all the breakthroughs published in the literature, it is crucial that we consider the concept of ‘water as a biomolecule’ (Ball, 2008) to avoid missing an essential part of knowledge for students and researchers in the future. Instead, the cytomatrix should be viewed as a cooperative system of supramolecular water-ion-protein complexes (Shepherd, 2006).

A major point in understanding water’s biophysical properties in the intracellular environment is its evolution in a very crowded space (Fayer, 2011). Reviewing its role in the subcellular structuring of a prokaryotic cell, Spitzer looked back to the pioneering studies conducted on marine species, and suggested viewing the physical and chemical interactions and processes that structure the cytoplasm through a ‘complex vectorial (bio)chemistry’ (Spitzer, 2011). Another consequence is that water entropy is maximized, with an impact on the cytoskeleton and gene expression (Li et al., 2022). Evidence that crowding provided a mechanism by which cells are sensitive to their volume change had previously been supported by experimental observations (Burg, 2000), a significant parameter for egg and embryo buoyancy in marine species, and thus their ecological behavior. Meanwhile, direct observation of the spatial distribution of water molecules inside a living cell has revealed different crowding environments in the nucleus and cytoplasm (Takeuchi et al., 2017).

Active fluxes of water and solutes of water play an essential role during cell shape changes and cell motility, emphasizing the role of hydraulic pressure in cell dynamics (Li et al., 2020). Cell volume regulation also involves ion/water transport systems, and the last two decades were characterized by tremendous insight into the role of aquaporins (Verkman, 2005; Papadopoulos et al., 2008). Besides the cytoskeleton, which had long been suggested as generating the driving force for cell migration, the water flow due to osmotic gradients generated by localized ion transport across the plasma membrane also contributes to this process (Morichita et al., 2019). Experimental evidence for anomalous diffusion of water in biological tissues, measured by nuclear magnetic resonance (NMR) has been extensively documented (Köpf et al., 1996), leading to the development of diffusion-weighted magnetic resonance imaging (DW-MRI), applied to cancer diagnosis and follow-up after treatment (Patterson et al., 2008). Interestingly, the role played by another technique, quasielastic neutron scattering (QENS), provided additional value in understanding the role played by water molecules in tumors before and after treatment with chemotherapy drugs (Martins et al., 2022).

As an echo to biophysical investigations on structured (bound) water in cell biology (Pouliquen et al., 2006a; 2006b), a long-distance effect for structured water was observed, related to an ordering of water molecules binding to specific sites on surfaces such as biological membranes, forming localized clusters corresponding to what is called today an ‘exclusion zone’ (Chen et al., 2012). This term of ‘structured water’, commonly used by numerous NMR investigators of water in biological systems from the 1970s to the 1990s, refers to the gel-like consistency of water in the intracellular environment, nicely described by Pollack, who argued for a ‘marriage between interface science and biology’ (Pollack, 2003). In this vision, one last crucial parameter of the problem is also represented by the strength of hydration, which is charge density-dependent (Collins, 1997). Meanwhile, improvements were made in the knowledge of the ‘water affinity’ of the different ions, another interesting question of interest common to both marine and biomedical sciences (Collins, 2019).

Investigations on water properties in membrane interfaces (Disalvo et al., 2008) have progressively led to seeing water as a structural and thermodynamic component of biomembranes (Disalvo et al., 2022). Given the impact of the crowding effect on the thermodynamics of metabolic reactions, the way water shapes proteins was recently documented, revealing its role in protein stabilization (Crilly et al., 2021). In parallel, water wires were shown to be critical for the functioning of membrane proteins (Paulino et al., 2020). Protein stabilization is also a key issue for preserving living cells and tissues. As different freezing approaches are currently used, the molecular interplay between cryoprotectants and water/ice were analyzed using molecular dynamics simulations in the context of protein stabilization and preservation of cell membranes (Weng et al., 2019). Water being replaced by sugars, the way in which extremotolerant organisms exploit sugars as desiccation protectants for preserving protein structure is another area undergoing intense investigations (Brom et al., 2023). Finally, biophysical studies of organisms living in extreme conditions revealed mechanisms for sensing water stress and the relevant signal transduction pathways involved in cellular responses to water-deficit stress (Caramelo and Iusem, 2009).

**FIGURE 1:**
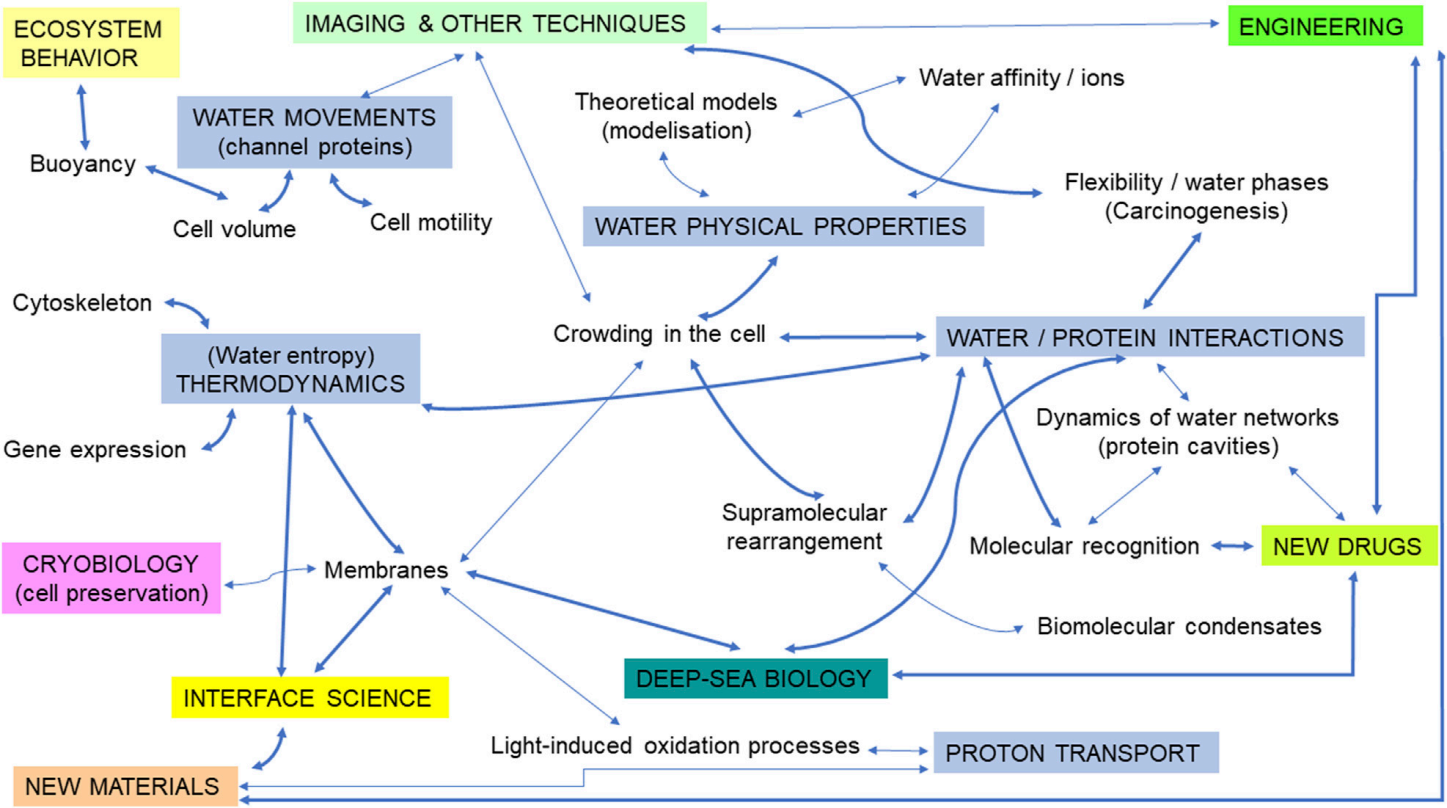
An illustration of the different topics corresponding to potential intellectual bridges at the interface between the biophysics of water in the cell/marine biology/cancer research. Subtopics relevant to water biophysics are illustrated in blue rectangles (uppercase letters). Thicker arrows represent suggestions for priority research fields likely to open innovative perspectives. Some connections between terms (symbolized by arrows) were voluntarily removed for clarity.

Here, starting with a survey of the most recent findings, we will examine questions relevant to both marine biology and cancer research (Figure 1), pointing to some potential intellectual bridges between them, likely to open interesting prospects for the future.

## 2 Recent breakthroughs on the biophysics of water in the cell

The mechanisms of water movements have received considerable attention. The role played by aquaporins in regulating migration-related processes was reviewed, highlighting their complex interrelationships with cell volume change, and signaling pathway activation (Smith and Stroka, 2023). Based on simulations of cellular water exchange, improved current physical diffusion models were proposed (Gardier et al., 2023). This is in line with parallel findings revealing vascular leakiness to circulating fluids, involving endothelial cell gap formation related to lamellipodia dynamics (Arce et al., 2023). The question of membrane water permeability was also updated through changes in membrane structure. For example, calcitriol, a unique component of the *Helicobacter pylori* membrane, facilitates water transport, crossing from one layer to another more easily than cholesterol (Cao et al., 2024).

Salinity or membrane composition also tune the interactions between biomolecular condensates and membranes, a reciprocal mechanism existing between water activity and supramolecular rearrangement, with protein secondary structure altering water dynamics in turn (Mangiarotti et al., 2023). The way biomolecular condensation of intrinsically disordered proteins controls water availability in cells was also investigated (De Souza and Stone, 2023; Watson et al., 2023). Additional findings improving our understanding of cellular condensates were the dissection of the role played by water-mediated interactions in a prototypical cellular condensate environment using long-timescale atomistic simulations (Brown and Potoyan, 2024). The difficulty with studying water structure within cells directly was overcome by demonstrating the power of the water bend-libration combination band using Raman spectral imaging. This revealed fascinating images of cellular water subpopulations within neuroblastoma cells (Ramos and Lee, 2023). The consequence of the crowded space on water activity was also studied in the mitochondrial matrix, revealing the impact of its changes on biochemical reactions (Bulthuis et al., 2023). Another case showed the impact of crowding and cellular interactions on *in vivo* folding of the three-helix bundle protein B in the cytoplasm (Russell et al., 2023). Finally, the way macromolecular crowding affected the enzymatic reaction provided by hydrolases, through changes in water structure, was detailed (Perillo et al., 2023).

A central question related to these investigations concerns thermodynamics. Earlier works questioning the thermodynamic explanation for water-protein interactions (Watterson, 1997), and how solid-state physics could help understand the behavior of hydrated proteins (Teeter et al., 2001), found some echo in recent publications. First, free energies analyzed for four hydrated globular proteins differing by their net charges revealed that water was most stable around anionic residues, and least stable near hydrophobic residues (Kalayan et al., 2023). As most biomacromolecules fold into chiral structures for their biological functions, how water molecules rearrange to minimize free energy at interfaces was shown, with achiral water molecules assembling in the first hydration shell of the protein into a chiral supramolecular structure with chirality transferred from the protein (Yan et al., 2023). Applied to ribonuclease A, an ultra-high-resolution x-ray analysis structure exhibited a refined model based on two times more water molecules interacting with the protein, with thermodynamic implications (Lisgarten et al., 2023).

More than 25 years ago, the unique properties of water molecules were recognized as being key players for protein-DNA interactions and molecular recognition (Robinson and Sligar, 1998). Their hope for further biochemical and biophysical work was recently accomplished when the thermodynamics of water networks in protein cavities was investigated in the context of rational drug design (Barros et al., 2023). The way water fine-tunes the 3D shape and dynamics of tumor-associated carbohydrate antigens was also nicely highlighted (Bermejo et al., 2018). Investigations into water-protein interactions reported the role of water molecules in the activation of G protein-coupled receptors (Hu et al., 2023). However, in the cell, water also serves as a substrate for proton transport. Accordingly, the role of water transport and proton release, for water molecules delivered to the catalytic center in photosystem II (PSII) was determined. This work led to an updated view of how ordered water molecules within the different channels contribute to catalyzing the light-induced oxidation of water into molecular oxygen (Doyle et al., 2023). In the same field, in cytochrome c oxidase, the order and molecular dynamics of protonation in its two distinct channels and the number of water molecules required for proton transport were deciphered (Gorriz et al., 2023). Finally, to improve our knowledge of the controlled diffusion of protons through transmembrane proteins, it was hypothesized that protons are conducted through dry apolar stretches by forming transient water wires (Kratochvil et al., 2023).

## 3 Lessons from the oceans

Given the extraordinary life diversity in the oceans, these findings could inspire engineering for the coming decades, for example, in the design of new materials for industry and healthcare. In the case of the *Photobacterium* genus, which includes psychrophile and piezophile species living in symbiosis with marine organisms, an interesting study highlighted the role of cosolvent-water interaction in the modulation of bacterial luciferase functionality (Lisitsa et al., 2023). The mechanism of light-driven water photooxidation of PSII isolated from the halotolerant green alga *Dunasiella salina*, revealed an unexpected level of conformational flexibility (Caspy et al., 2023). In channel rhodopsins, the way the timing of the proton transfer was tightly controlled was investigated, showing how the number and location of water molecules close to the proton transfer groups had an impact on the proton transfer pathways (Adam and Bondar, 2018).

For organisms living in the deep sea, a fundamental question concerns the effect of high hydrostatic pressure and how they adjust the volume changes of their biochemical reaction in cellulo (Oliva et al., 2020). Adapting to extreme environmental conditions was initially analyzed in terms of the conformational stability of proteins (Jaenicke and Závodszky, 1990). This question, later extended to the sub-seafloor and continental subsurface, led to investigations into the mechanisms driving molecular adaptation, through the stabilization effect of small molecules known as piezolytes, among which the most potent is trimethylamine *N*-oxide (TMAO). Using Fourier transform infrared (FTIR) spectroscopy combined with electronic-structure-based computer simulations, pressure-induced changes were connected to a locally enhanced H-bonding network at high compression (Imoto et al., 2016). The effect of TMAO was then attributed to its large dipole moment, making it possible to form strong interactions with water molecules by forming H-bonds with at least three of these molecules, resulting in preferential hydration of the protein surface (Kamali et al., 2022). Recently, this protective effect was extended to high temperature TMAO molecules binding very specific amino acids on the protein surface while other molecules ‘in a shell further away from the protein herd water molecules to enhance protein stability’ (Boob et al., 2023). Finally, in nucleic acids, TMAO rescued the shift produced by high pressure in the conformational equilibrium of a DNA hairpin into the open, unfolded state (Patra et al., 2018).

**FIGURE 2:**
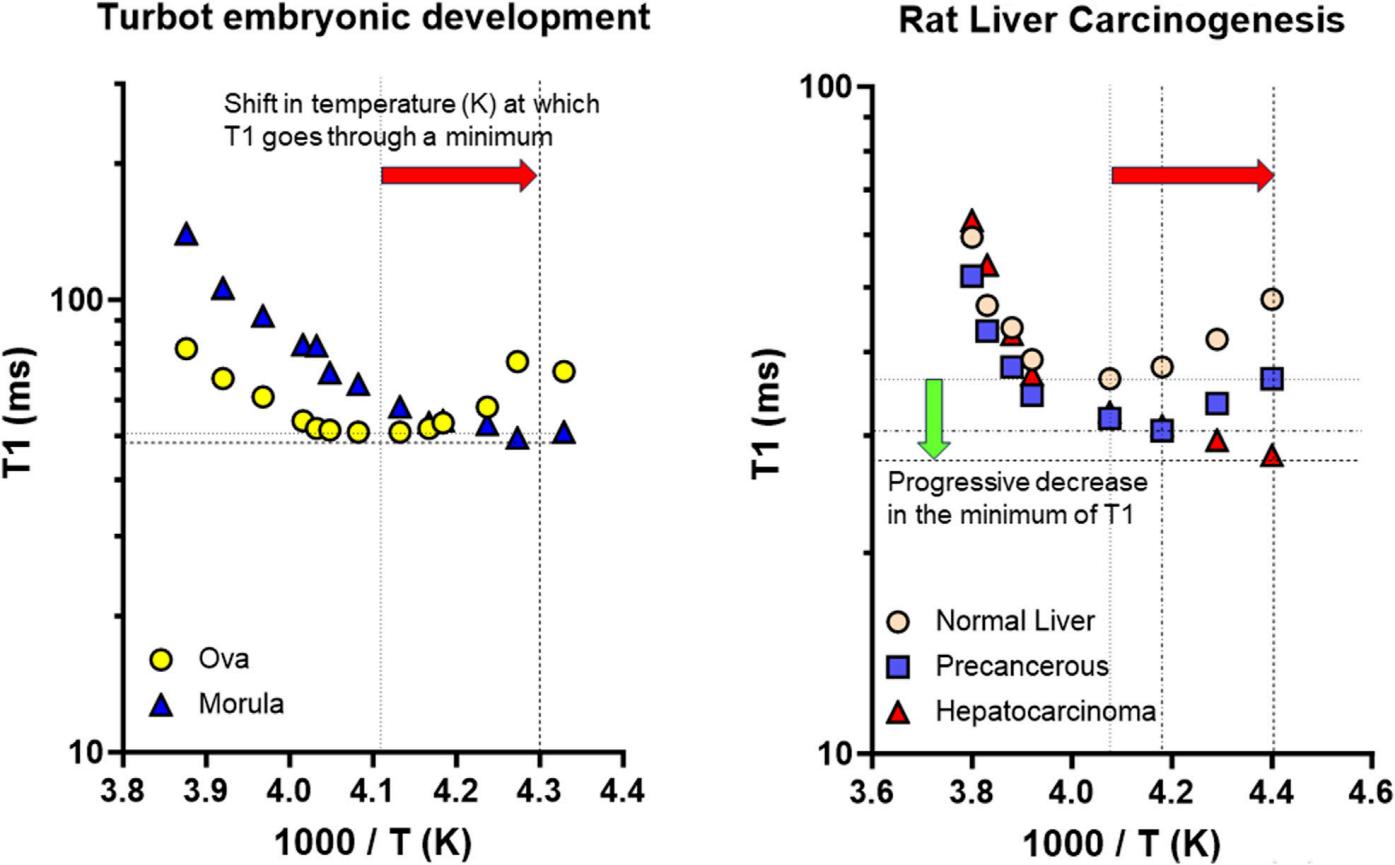
Examples of ^1^H NMR investigations into the biophysical properties of structured (bound) water in experimental models relevant to marine biology and cancer research. Adapted from Pouliquen, D. et al. *Comp. Biochem. Physiol. B* (1998) 120, 715–726. doi: 10.1016/S0305-0491(9,810,067–6) and Pouliquen D., et al. *Anticancer Res.* (1993) 13, 49–56, with permissions. Both models presented a shift (horizontal red arrow) in the temperature corresponding to the minimum of the spin-lattice relaxation time (T1), meaning correlation times for the rotational motion τ_R_ of unfrozen water molecules were decreased during the early development of turbot (*Psetta maxima*) embryos, and during rat liver carcinogenesis. A common increase in the activation energy for the rotational motion *E*_*R*_ occurred in parallel. However, the two evolutions differed by the decrease (green vertical arrow) in the minimum of T1, only observed in the second case, which meant additional changes in the cross-relaxation parameters between water and macromolecular protons. Correlation times were calculated from the procedure described in the supplementary text of Pouliquen et al. (2006a) (https://www-nature-com/articles/4401731#Sec18), Pouliquen, D., et al. *Cell Death Differ.* (2006) 13, 301–310. doi: 10.1038/sj.cdd.4401731.

TMAO was found early in tissues from marine organisms (Yancey et al., 1982). Although initially debated, the hypothesis that TMAO was adaptively regulated with depth in deep-sea teleosts progressively gained support (Samerotte et al., 2007), and the highest TMAO contents in teleost marine fishes was found in *Notoliparis kermadecensis*, the deepest known fish in the southern hemisphere (Yancey et al., 2014). Paul H. Yancey also mentioned that, although ‘TMAO can effectively counteract many inhibitory effects of hydrostatic pressure on numerous proteins’, ‘for vertically migrating marine animals, hydrostatic pressure stress responses are even more poorly characterized’ (Yancey, 2020). Interestingly, his investigations connect with previous studies based on the combination of ^1^H-NMR relaxometry and spectroscopy on the evolution of bound (‘structured’) water during the early development of turbot (*Psetta maxima*) (Pouliquen et al., 1998) (Figure 2). Our results could be reinterpreted in the light of reports confirming the presence of TMAO in extracts of juvenile turbot (Hoerterer et al., 2023), and in oocytes of the common carp (*Cyprinus carpio*), showing increased TMAO levels with post-ovulation time (Hajirezaee et al., 2021). The considerable variability we observed in the spin-lattice relaxation times of structured (unfrozen) water could be related to TMAO content changes, connected to its role in stabilizing protein structure during yolk protein proteolysis post-ovulation. Improvements in our understanding of the biophysical/biochemical parameters affecting fish egg hydration, and how piezolytes act, are also crucial in relation to buoyancy changes during their early embryonic development. In this field, the discovery of new aquaporins has provided insight into the molecular basis of the production of viable eggs (Cerda, 2009). The multiple functions these channels have was extensively reviewed (Cerda et al., 2017). Many efforts were also made to develop the theoretical basis for buoyancy variations in fertilized eggs, for example, applied to the Atlantic cod (*Gadus morhua*) (Jung et al., 2014). The case of vertical distribution was examined for marine fish eggs; however, its importance is crucial across species and for many ecosystems given the impact of climate change (Sundby and Kristiansen, 2015). Connected to lipid composition changes in the lipid sac and membranes of marine planktonic copepods, regulating buoyancy determines their seasonal life cycle, and in particular their vertical migration (Pond et al., 2014).

One last issue concerns TMAO interactions with lipid membranes. A gel-to-fluid phase transition was observed, shifting to higher temperatures with increasing TMAO concentration, leading to a drastic water loss in the interlamellar space of fully hydrated multivesicular lipid assemblies (Manisegaran et al., 2019). For some microorganisms living in deep-sea hydrothermal vents, the biophysical properties of archaeal membranes also revealed lower water permeability compared with that of *n*-acyl phospholipids, while remarkably, macrocyclization improved the membrane barrier to water (Dannenmuller et al., 2000). The liquid-crystalline state that characterized their membranes, in addition to their low permeability, appeared to be an adaptation for living in a wide range of econiches, from cold ocean water to high temperatures and pressure in hydrothermal vents (Chugunov et al., 2014).

## 4 Discussion

^1^H-NMR investigations of marine fish eggs/embryos, and normal/neoplastic tissues identified changes in water state, revealing a common increased dynamics of structured water in early development and carcinogenesis. However, the normal-to-cancer transition involved specific additional changes in the cross-relaxation between water and macromolecular protons (Figure 2; Pouliquen et al., 1993; 1995; 2001). These findings agree with differences observed in heat capacity in tumorous vs. normal tissues (Vaupel and Piazena, 2022). Using QENS, considerable diversity was also observed in the flexibility of the different types of intracellular water in normal and cancerous cells (Marques et al., 2020), while MR elastography highlighted its link with tumor fluidity (Streitberger et al., 2020). Conversely, a fluid-to-solid transition characterized proliferative cells becoming dormant (Munder et al., 2016). Differences in composition analyzed through simulations also revealed a shift in electron density of water in line with the lower stability observed in cancer vs. normal membranes (Elfiky et al., 2023). However, other topics could benefit from transdisciplinary bridges between marine biology and cancer research, and *vice versa*. First, the role of TMAO in tumorigenesis, initially related to the anaerobic metabolism of Enterobacteriaceae (Barrett and Kwan, 1985), is increasingly questioned, especially for colon cancer (Duizer and de Zoete, 2023). However, TMAO directly drives an immunostimulatory phenotype in macrophages, supporting T cell responses, and reducing pancreatic ductal adenocarcinoma burden (Mirji et al., 2022). Accordingly, in triple-negative breast cancer, TMAO activated CD8^+^ T cell-mediated immunity by inducing pyroptosis in tumor cells (Wang et al., 2022). This topic thus requires much more investigation. Secondly, some aquaporins are prospective biomarkers of prognostic significance in prostate cancer (Kushwaha et al., 2023), the role of aquaporin five in lung cancer beingalso questioned (Jaskiewicz et al., 2023). Water exchange through aquaporin-4 being measured by MRI, transmembrane water-efflux rates are a biomarker of proliferative glioma (Ruan and Keshari, 2022). MRI also allows to investigate damage to the myelin sheath, making it possible to study the different water pools in complex macromolecular environments (van der Weijden et al., 2023). This, like MR microscopy (Pooh et al., 2011), chemical exchange saturation transfer (CEST) MRI (Maralani et al., 2023), or DW-MRI for determining microscopic tumor spread (Shusharina and Nguyen, 2023), could increasingly benefit to marine biology, as shown recently (Chanet et al., 2023; Sauer et al., 2023; Gerussi et al., 2024).

In conclusion, an increasing number of molecules isolated from oceans show interesting properties in oncology/immunology. Integrating the complex role of water molecules in the cell, and its changes during neoplastic transformation are expected, for better understanding molecular recognition and optimizing engineering-based drugs and materials. All questions relevant to water entropy, protein interactions, supramolecular rearrangement and membrane biology are also a huge domain, which could lead to many innovations in both disciplines.

## Data Availability

The original contributions presented in the study are included in the article/Supplementary Material, further inquiries can be directed to the corresponding author.
